# Scraps

**Published:** 1890-03

**Authors:** 


### SCRAPS

PICKED UP BY THE ASSISTANT-EDITOR.

---

**Good for Prescriptions.**—School-teacher to anxious parent: "Your son is bright, intelligent, and getting along well in everything but handwriting." Parent: "That 's all right; his handwriting doesn't matter. I am going to make a doctor of him."—*Cincinnati Lancet-Clinic.*

**Festina Lente.**—Invalid: "I have been here at these springs, doctor, six weeks, and I don't see that the water has had the slightest effect." Dr. Candid: "You must have patience. There was a man here last season who didn't die until after he had been here two months."

**An Impatient Child.**—A case of Cæsarean section was down on the bills at one of our city clinics the other day; but the child would not wait, and came into the world by the usual route before the clinic day arrived.—*Medical Times and Register* quoted in *The Obstetric Gazette.*

**Professional Advertising.**—Amongst the younger medical men of this neighbourhood there is a keen observer who suggests that doctors who are desirous of being specially obtrusive might borrow an idea from the Railway Carriers, and affix to their carriages some such notice as this: "Upon request the driver of this vehicle will stop, and the doctor inside will, upon payment in advance, attend to any message that may be given."

**Honour to whom Honour is Due.**—The following "Birth Notices" appear in the *Cincinnati Enquirer*:—"FLAMIN.—Saturday, 9th inst., at 8.15 a.m., to the wife of D. W. Flamin, at College Hill, a ten-pound boy. Thanks to Dr. Wallingford, of Cincinnati." "GALLION.—June 5th, to Mrs. MONA GALLION, of Liberty Street, a nine-pound girl. Thanks to Dr. Wallingford." This is, doubtless, an effective way of advertising Dr. Wallingford's peculiar qualifications; but it would seem to us a little strange if Messrs. Flamin and Gallion should be content to allow the doctor all the credit for the happy occurrences.—*The Medical Age.*

**Une Consultation.**—L'annonce de la fermeture de la chasse a soulevé une nouvelle facétie à l'adresse de ceux de nos confrères qui cumulent les fonctions de disciple d'Esculape et de saint Hubert. En un jour de chasse, deux d'entre eux tirent un même perdreau avec tant d'ensemble que les deux coups de fusil n'en font qu'un. Le gibier tombe; les deux chasseurs se précipitent. "C'est moi qui l'ai tué," dit l'un. "C'est moi," dit l'autre. "Funeste effet d'une consultation," s'écrie l'amphitrion chez qui on chassait; "la mort du patient! Mais avouez, Messieurs, que, s'il agissait de la mort d'un client, vous mettriez moins d'empressement à vouloir vous l'attribuer."—*L'Union médicale.*

**Posture in Labour.**—This question, which was most interestingly treated by Dr. Engelmann in his *Labor among Primitive Peoples* in 1882, has been discussed by Dr. King in the *American Journal of Obstetrics*, No. 4, 1889. *The Medical Record* says that some of his "conclusions, if true, reflect little credit upon the modern obstetric art. If 'the normal mechanism of labor is still imperfectly understood,' it argues extraordinary dulness on the part of physicians who have been delivering babies since medical science began. Furthermore, as over ninety per cent. of labors are normal, and take place in almost precisely the same way, it seems as though obstetricians should be able to tell the woman which would, with the greatest probability, be the proper position for her to adopt. Professor King, it seems, would leave the unhappy parturient uncertain whether she should squat like a Kaffir, hang from a rope like an Apache, kneel like a Mexican, hang to a pole like a Blackfoot squaw, stand like the Ceramites and ladies of the Nile, lie on the stomach like the Creeks, on the side like the English, or on the back like the Americans. It seems but fair that modern obstetrics should give parturient ladies less postural latitude than is implied in Dr. King's conclusions, which, we take it, are held to apply to the second stage of labor."

**Antisepsis in Excelsis.**—The fear of microbes, which is rapidly becoming a dominant factor in modern life, has lately received a curious illustration at Nordhausen, a small town in Prussian Saxony. A municipal edict has been issued that all barbers, hairdressers, and "tonsorial artists" generally shall, under legal penalties, well and truly disinfect their instruments every time after they have been used. This is, no doubt, a step in the right direction; but it is to be hoped that these enlightened councillors will not stop here in their career of sanitary progress. As so many doctors in Germany are at present perforce in the ranks of the "unemployed," why should they not be invited to revive in their own persons the defunct order of "barber-surgeons"? The idea of the Nordhausen municipality could then be carried out in a manner befitting its importance. Shaving might be performed as a surgical operation with all due antiseptic rites and ceremonies, the chin being afterwards dusted with "double cyanide" instead of violet powder. The carbolic spray would, of course, replace the orthodox "blow" of bay rum. When "Haircutting on Listerian Principles" meets the eye on every hairdresser's signboard, the illustrious founder of the antiseptic system may sing his *Nunc dimittis* with the proud consciousness that he has not lived in vain.—*The London Medical Recorder.*

**An Unappreciative Physician.**—Under the title "Good Books and Periodicals *versus* Ignorance and Laziness—a Rich Letter," the *Texas Health Journal* says: "The indifference of some at the current medical and sanitary literature of the age is passingly strange. It is a problem hard to solve. These reflections result from a recent incident incomprehensible in its immensity. A subscriber returned this *Journal* with the following soul-stirring revelation: 'Stop the Medical Health—am over red now—all I knead is treatment—got to big a library any how—besides I already take the theripudic gazet—dident subscribe for your *Journal* know how,—M.D.' We are confident this man is over 'red'—but just how 'red' we have no means of ascertaining—and that he thoroughly 'kneads' his treatment—blue mass especially. Being already over-stocked with the 'theripudic gazet,' and 'to big a library,' he 'dident' 'knead' the *Journal* 'know how'—therefore we feel indisposed to press our claims, lest the gentleman become overwhelmed with pure 'medical health.' Regretfully we leave him to the peaceful enjoyment of his erudition and peculiar vernacular. So long as such men are entrusted with human lives the cause of a high mortality in some localities may, with some degree of certainty, be surmised."—*The Medical and Surgical Reporter.*

In his reasons for discontinuing his *Journal*, the Texas doctor is not without his British representative. Equally funny excuses have been alleged by some who at one time subscribed to this *Journal*. May I suggest to existing subscribers that, as this is the first number of a new volume, the time is an excellent one for inducing their friends to take in the *Journal?*

